# A Reference Hand-Book of the Medical Sciences

**Published:** 1903-09

**Authors:** 


A REFERENCE HAND-BOOK OF THE MEDICAL SCIENCES.—Embracing the Entire Range of Scientific and Practical Medicine and Allied Science. By various writers. A new edition, completely revised and rewritten. Edited by Albert H. Buck, M. D., New York City. Volume V. Illustrated by chromo-lithographs and 576 half-tone and wood engravings. Pages, 873. New York: Wm. Wood & Co. 1903.

This volume is an especially valuable one. It contains a symposium of articles by the leading alienists of the country, constituting a really very complete treatise on the subject of insanity. The kidney diseases and liver diseases are similarly treated in groups; also the affections of the larynx, and a large proportion of the diseases affecting the lungs. The articles relating to diseases of the eye are scattered throughout the entire series of eight volumes, and the present volume contains several important ones. A large number of the leading laryngologists of the United States and Canada have taken part in the preparation of the articles relating to the larynx. This group, taken in connection with the symposium of ear articles in volume four, and the similar collection of articles on the nasal cavities to appear in volume six, makes one of the most exhaustive treatises on the affections of the ear, nose and throat that has ever been published in the English language.

As regards the isolated articles, there are not a few which alone are good enough to make the reputation of the book. Among these may be mentioned: Dr. Theodore Janeway's article on the "Leukæmias;" Professor Ward's article on "Mosquitoes in Relation to Human Pathology;" Dr. Schauffler's very practical article on "Malarial Diseases;" Professor Osborn's article on "Parasitic and Poisonous Insects;" Professor Councilman's article on "Inflammation;" Dr. Robert Lovett's article on "Chronic Diseases of Joints;" Dr. Benjamin Tilton's article on "The Surgical Treatment of Diseases of the Liver;" Dr. Arthur I. Cabot's article on "Lithotapaxy and Lithotomy;" Dr. Oliver Ornsby's article on "Leprosy;" Dr. E. T. Brackett's article on "Lateral Curvature of the Spine;" Dr. George Dobbin's article on the "Management of Normal Labor;" Prof. Joseph W. Warren's article on the "Physiology of the Reflexes;" Dr. Arthur Shrady's article on "Tuberculous Diseases of the Lungs;" Dr. Dawbarn's article on the "Starvation of Malignant Growths;" Prof. Lafayette B. Mandell's article on the "Metabolic Processes;" Dr. Robert Lewis' article on "Mastoid Operations," etc.

The above represent only a portion of the treasures which this volume contains. Very many of equal importance might be enumerated if space permitted. It is doubtful if any book published in this country or in Great Britain ever contained so much information useful to medical men of all sorts—scientific inquirers, busy family practitioners, or men who devote themselves to some special department of medical practice—as does volume V.

It is clear that no practitioner can afford to be without the Reference Hand-Book, and it is not exaggeration to say that there is not a set of books published that is superior to it. T. J. B.

